# Smartphone / smartwatch-based cuffless blood pressure measurement : a position paper from the Korean Society of Hypertension

**DOI:** 10.1186/s40885-020-00158-8

**Published:** 2021-01-25

**Authors:** Hae Young Lee, Dong-Ju Lee, Jongmo Seo, Sang-Hyun Ihm, Kwang-il Kim, Eun Joo Cho, Hyeon Chang Kim, Jinho Shin, Sungha Park, Il-Suk Sohn, Wook-Jin Chung, Sung Kee Ryu, Ki Chul Sung, Juhan Kim, Dae-Hee Kim, Wook Bum Pyun, on behalf of the Korean Society of Hypertension

**Affiliations:** 1 Seoul National University College of Medicine, Seoul, South Korea; 2 Department of Electrical and Computer Engineering, College of Engineering, Seoul National University, Seoul, South Korea; 3 Division of Cardiology, Department of Internal Medicine, Bucheon St. Mary’s Hospital, College of Medicine, The Catholic University of Korea, Seoul, South Korea; 4 Division of Geriatrics, Department of Internal Medicine, Seoul National University Bundang Hospital, Seongnam, South Korea; 5 Division of Cardiology, Department of Internal Medicine, Yeouido St. Mary’s Hospital, College of Medicine, The Catholic University of Korea, Seoul, South Korea; 6 Department of Preventive Medicine, Yonsei University College of Medicine, Seoul, South Korea; 7 Division of Cardiology, Department of Internal Medicine, Hanyang University Medical Center, Seoul, South Korea; 8 Division of Cardiology, Severance Cardiovascular Hospital and Severance Cardiovascular Hospital and Integrated Research Center for Cerebrovascular and Cardiovascular Diseases, Yonsei University College of Medicine, Seoul, South Korea; 9 Division of Cardiology, Department of Internal Medicine, Kyung Hee University at Gangdong, Seoul, South Korea; 10 Division of Cardiology, Department of Internal Medicine, Gachon University Gil Medical Center, Incheon, South Korea; 11 Division of Cardiology, Department of Internal Medicine, Nowon Eulji Medical Center, Eulji University, Seoul, South Korea; 12 Division of Cardiology, Department of Internal Medicine, Kangbuk Samsung Hospital, Seoul, South Korea; 13 Division of Cardiology, Department of Internal Medicine, Chonnam National University Hospital, Gwangju, South Korea; 14 Department of Cardiology, Asan Medical Center, College of Medicine, Ulsan University, Seoul, South Korea; 15 Division of Cardiology, Department of Internal Medicine, Ewha Womans University Seoul Hospital, Seoul, South Korea

**Keywords:** Blood pressure, Measurement, Hypertension, Smartphone, Plethysmography

## Abstract

Smartphone technology has spread rapidly around the globe. According to a report released by the Korea Information Society Development Institute, about 95% of Koreans aged more than 30 years old owned smartphones. Recently, blood pressure (BP) measurement using a photoplethysmography-based smartphone algorithm paired with the smartwatch is continuously evolving. In this document, the Korean Society of Hypertension intends to remark the current results of smartphone / smartwatch-based BP measurement and recommend optimal BP measurement methods using a smartphone device. We aim to increase the likelihood of success in implementing these new technologies into improved hypertension awareness, diagnosis, and control.

## Background

Higher blood pressure (BP) levels are associated with an increased risk of cardiovascular disease (CVD) and mortality [1]. Moreover, the probability of progression to hypertension (HTN) and the risk for a cardiovascular (CV) event was higher in the elevated BP or prehypertension groups than those in the normal BP group [2]. Although the overall management of HTN has improved over the past few decades, the rates of awareness, treatment, and control are recently stagnant even in the developed countries [3].

Accurate measurement of BP is essential for the diagnosis, treatment, and prognostication of individuals with hypertension [4, 5]. In the office or in the clinic, the auscultatory method of measuring BP using a stethoscope is still considered as a standard one. BP measuring devices include a mercury sphygmomanometer, an aneroid sphygmomanometer, and an electronic sphygmomanometer. The mercury sphygmomanometer has been replaced by an electronic or aneroid sphygmomanometer because of environmental mercury pollution. The importance of BP measurement outside the office has continuously empathized in clinical practice. Out-of-office BP measurement provides better prognostic information than office BP measurement alone [6]. Moreover, the self-measurement of BP can improve HTN awareness and also adherence [7].

Mobile technology has been widely adopted all over the world. Today, more than 5 billion people use mobile devices, and over a half of these are smartphones. According to a report by the Korea Information Society Development Institute in 2018 [8], 37.8% of Koreans aged more than 70 years old owned smartphones in 2018, comparing 3.6% in 2013. The number of smartphone users increased over the last 5 years, with the number for people in their 60s rose from 19 to 80.3%, 50s from 51.3 to 95.5%, 40s 81.3 to 98.4%, and 30s 94.2 to 98.7%. Indeed, the percentage of adults who own smartphones is higher in South Korea than in any other developed countries (95%), including Israel (88%) and the Netherlands (87%) [9]. Even before the emergence of smartphones, various wearable devices were developed to estimate BP by photoplethysmographic sensors and signal analysis [10]. However, shortly after the introduction of the conventional smartphone in 2007 (iPhone) and 2008 (Android), it was soon discovered that the smartphone camera could be used as a photoplethysmographic sensor to obtain a signal waveform [10]. Recently, BP measurement using ‘cuffless’ photoplethysmography-based smartphone algorithm paired with the smartwatch is continuously evolving. Indeed, recent smartwatch devices fulfilled the requirement for a medical device in the International Organization for Standardization (ISO) 81,060–2:2018 Non-invasive sphygmomanometers [11].

In this document, the Korean Society of Hypertension intends to remark the current results of smartphone / smartwatch-based BP measurement and recommend optimal BP measurement using a smartphone device. We aim to describe the current limitations and requirements to guide the successful implementation of these new technologies for improving HTN awareness, diagnosis, and control.

## Part 1. Current results of smartphone-based blood pressure measurement

### Data source and searches

The Pubmed database was systemically searched for studies published from January 2010 through July 2020. Our Pubmed search query was ‘smartphone’ or ‘smart phone’ and ‘blood pressure’, explicitly focusing on title/abstract. We included every paper until July 2020. Not only English but also every paper in other languages were included. Also, all references from selected papers were therefore assessed for relevant studies.

Studies retrieved from the initial database search were examined by predetermined selection criteria. Inclusion criteria were as follows: (1) related to direct BP measurement, and the measurement method is different from the conventional method; (2) smartphone is used as a BP measuring device; (3) original paper that conducted experiments on real people. The exclusion criteria were as follows: (1) an application (App) encouraging BP measurement; (2) principle of oscillometric or sphygmomanometer are used as a measurement method; (3) Self-manufactured equipment; (4) Review papers. Observational studies, study protocols and designs, studies with an abstract presentation, and duplicates were all excluded.

### Search results

**Fig. 1:**
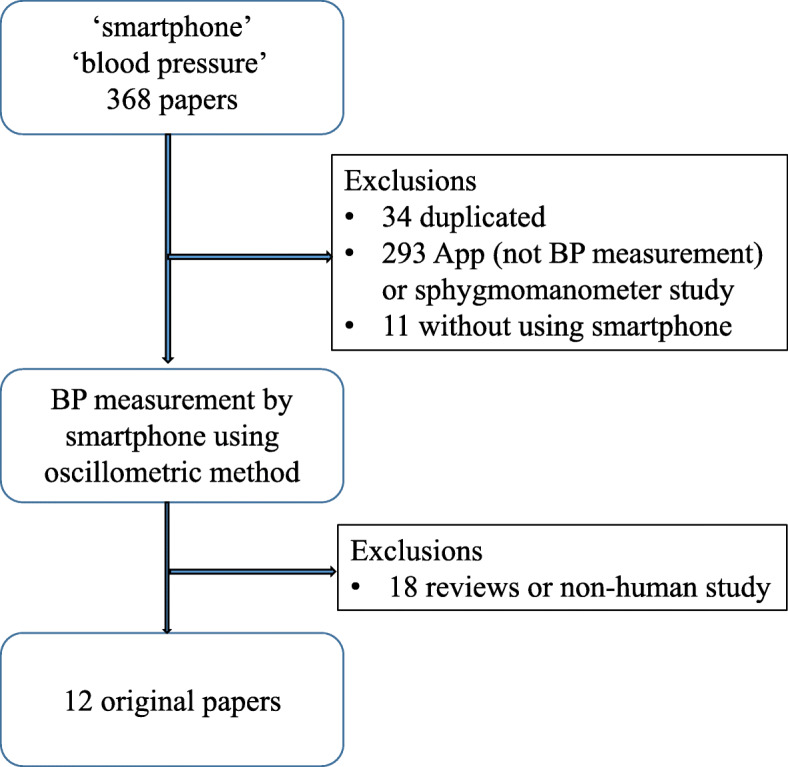
Study selection

A total of 368 papers were initially identified from the Pubmed database; 34 duplicated papers were excluded. The remaining 334 studies were then screened by two independent cardiologists. We excluded 293 papers because of the low relevance of the title and abstract of this paper. A further 11 papers were excluded because they did not evaluate the smartphone as a BP measurement device. After reviewing the full text of the remaining 30 papers, 18 papers were excluded due to reviews or non-human studies. All of the 12 papers were included in a narrative synthesis. A flow diagram of the selected studies is shown in Fig. 1.

### Study characteristics

The characteristics of the 12 papers are shown in Table 1. Six studies were conducted in general subjects with normal BP, and the other six studies evaluated in subjects with varying BP ranged. Seven studies used iPhones as a BP-measuring device, three for Galaxy, and the rest for Google Pixel, Android, and Nexus, respectively. Four studies used a sphygmomanometer as a comparison method, while eight used the oscillometric method, and one used a finger sensor.

**Table 1:** Summary of smartphone / smartwatch-based cuffless blood pressure measurement

Year	Authors	Subjects	Type of subjects	Device	Comparison (Measuring location / device)	Mean ± SD (mmHg)	
						SBP	DBP
2013	Chandrasekaran, et al. [12]	500	Healthy subjects	Nexus One	Brachial cuff / Mercury sphygmomanometer	±2.4473 (95% CI)	±1.7073 (95% CI)
2014	Visvanathan, et al. [13]	156	Variable subjects	Iphone4	Brachial cuff / ETCOMM HC-502	0.78 (F-score)	0.80 (F-score)
2016	Plante, et al. [14]	85	Variable subjects	iPhone 5 s, 6	Brachial cuff / Omron 907, 907XL	12.4 ± 10.5	10.1 ± 8.1
2016	Gaurav, et al. [15]	3000	Healthy subjects	galaxy note 5	Brachial cuff / Mercury sphygmomanometer	0.16 ± 6.85	0.03 ± 4.72
2016	Alexander, et al. [16]	100	Healthy subjects	iPhone 5 s	Brachial cuff / Welch Allyn Propaq CS Monitor	−0.6 ± 12.8	+ 7.2 ± 9.2
2016	Gao, et al. [17]	78	Healthy subjects	Android phone	Brachial cuff / A&D UA-767PBT	5.1 ± 4.3	4.6 ± 4.3
2018	Raichle, et al. [18]	32	Variable subjects	Iphone 4 s	Brachial cuff / Omron-HBP-1300	5.0 ± 14.5	NA.
2018	Wang, et al. [19]	7	Variable subjects	Google pixel phone	Brachial cuff / Microlife BP3NA1-1x	N.A.	4.8 ± 4.7
2018	Chandrasekaran, et al. [20]	20	Healthy subjects	iPhone X	Brachial cuff / Omron BP786	−4.0 ± 11.4	− 9.4 ± 9.7
2018	Dey, et al. [21]	205	Variable subjects	Galaxy S6	Brachial cuff / Mercury sphygmomanometer	6.90 ± 9.00	5.00 ± 6.10
2018	Matsumura, et al. [22]	49	Variable subjects	iPhone 6 s	Brachial cuff / NISSEI, DS-S10	0.67 ± 12.7	0.45 ± 8.6
2019	Luo, et al. [23]	1328	Healthy subjects	iPhone 6+	Finger sensor / CNAP 500	0.39 ± 7.3	−0.20 ± 6.00

Two studies satisfied the Association for the Advancement of Medical Instrumentation (AAMI) criteria [24], which indicates that only these studies can be used as a valid BP measuring instrument. The other ten studies did not meet the criteria. In a paper on various BP studies, the more the BP values deviated from normal, the larger the error was. In addition, all studies have stated that further studies are warranted on the limitation of the insufficient number of samples and how accurate results will be obtained when this is done in real human subjects.

In the case of the early developmental period implementing smartphones as BP measuring devices by using the heartbeat and photoplethysmographic wave analysis combined with the computerized algorithm, the accuracy was between 95 ~ 100%. However, there were large fluctuations depending on the method of measurement [12]. Therefore, the measurement results were presented as BP ranges rather than actual BP values. In that case of describing the accuracy by BP range, F-scores of 0.78 and 0.8 {systolic BP (SBP) and diastolic BP (DBP)} were higher than using actual values [13].

Afterward, many studies have been conducted to use smartphones as BP measuring devices. However, there were no satisfactory results proving consistency with the standard BP measured by standard brachial BP measured by sphygmomanometers satisfying the AAMI standard [14, 15, 17–19].

However, recent studies have shown much-improving data by applying ubiquitous models or linear polynomial equation [21, 22]. Finally, the proof-of-concept study applying an iPhone camera sensor for BP monitoring via the oscillometric finger pressing method showed bias and precision errors of − 4.0 and 11.4 mmHg for SBP and − 9.4 and 9.7 mmHg for DBP [20]. These errors were near the finger cuff device errors, which is FDA-cleared for measuring brachial BP [25, 26]. Furthermore, this smartphone-based BP measurement using transdermal optical imaging technology method results in normotensive adults fall within 5 ± 8 mmHg of reference measurements, which satisfied a key accuracy threshold bias and SD when testing proceeds according to the AAMI standard [23, 24].

There are two critical concerns about BP measurement using a smartwatch device. One is that the accuracy of the photoplethysmographic sensor-based BP measurement is not validated. The existence of surrounding near-infrared (NIR) light sources could disturb accurate measurement [10]. Another problem is that there is low reliability of wrist BP because of an error in home self-measurement at the wrist despite appropriate training [27]. Although the data were from the cuff-inflation method of wrist BP, an appropriate static position is difficult in wrist measurement.

In summary, recent studies of BP measurement using a smartwatch device reported acceptable accuracy within 5 ± 8 mmHg of reference measurements, which satisfied a key accuracy threshold bias and SD when testing proceeds according to the AAMI standard. However, there is concern of wrist BP self-measurement at home even despite appropriate training.

## Part 2. Recommendations of blood pressure measuring method using smartwatch device

In order to use smartwatches as BP measuring device, it is mandatory to calibrate BP inputting the user’s BP values obtained by the standard BP monitoring device regularly. This calibration process might have the ancillary benefit to increase the awareness of BP. During the calibration process, it is recommended to measure BP by three times at least 2 min apart. Also, when the user changes the wrist wearing a smartwatch, the calibration process should be repeated.

The major issue in the calibration process is the difference in BP between the two arms. The mean inter-arm difference was 3.3 mmHg for SBP and 2.0 mmHg for DBP in extensive epidemiologic studies [28]. An increased inter-arm SBP difference (≥ 10 mmHg) is found in 5–10% of the population, associated with a future increase in SBP or progression to HTN [29]. Therefore, if the calibration was performed based on the BP values measured in the opposite arm, this can cause at least 3 mmHg error, which cannot be corrected by any internal calibration mechanism.

In the clinical trial, the research coordinators could help the subjects calibrate in the opposite arm while measuring BP using a smartwatch device. In this setting, simultaneous calibration using the opposite arm can minimize the temporal variation of BP. However, in the real world situation, the user cannot calibrate simultaneously without assistance, and then there is no benefit using the opposite arm for the calibration. Therefore, the experts recommend calibrating the smartwatch device using the same arm measurement of BP after acquiring photoplethysmography-based pulse signals in the smartwatch sequentially. It is crucial to acquire acquiring photoplethysmography-based pulse signals first because upper arm compression by cuff can influence the pulse signals by hyperemia [30]. In the American Heart Association BP measurement guideline [31], separate BP measurement repeated by 1–2 min is recommended. Venous congestion or hyperemia has traditionally been thought to affect the BP measurement results, especially when the Korotkoff method is used. However, in recent reports, very short time intervals between readings did not produce different values from conventional intervals when oscillometric devices were used [32].

The recommendation of the traditional cuff-based BP measurement method should be commonly applied to the smartwatch device, especially if the BP value was used for the diagnosis and the treatment of HTN^4^. The overview of proper seated BP measurement using a smartwatch device was summarized in Table 2.

**Table 2:** Overview of properly seated blood pressure measurement using smartwatch device

Critical steps for proper BP measurements	Specific introductions
Step 1: Properly prepare the position and the autonomic BP measurement device for the calibration	1. Have the patient relax, sitting in a chair with feet flat on the floor and back supported. The patient should be seated for 3–5 min without talking or moving around. Arm and wrist should be dry and free of excessive perspiration or skin lotion.
	2. The patient should avoid caffeine, exercise, and smoking for at least 30 min before measurement.
	3. Ensure that the patient has emptied his/her bladder.
	4. Neither the patient nor the observer should talk during the rest period or the measurement.
	5. Sit in the chair with the back well-supported, legs uncrossed, and feet flat on the ground. Support the user’s arm (e.g., resting on a desk). The patient should not be holding his/her arm because the isometric exercise will affect the BP levels. During the measurement, the user should breathe normally, avoiding deepen or slow down the breath.
	6. Use an upper-arm, cuff-based BP monitoring device validated and ensured that it is calibrated periodically.
	8. Use the correct cuff size such that the bladder encircles 75–100% of the arm.
Step 2: Calibration	1. Wear the smartwatch on the wrist, not too tight.
	2. Position the middle of the cuff on the patient’s upper arm (wearing smartwatch) at the level of the right atrium (midpoint of the sternum).
	3. Acquire the pulse wave by the smartwatch
	4. Acquire the reference blood pressure by the upper-arm, cuff-based BP monitoring device. At the first measurement, record BP in both arms. If the systolic blood pressures between two arms are different by 10 mmHg, visit the hospital and check the cause of the inter-arm difference.
	5. Then, enter the cuff-based blood pressure monitor reading on the smartphone. Repeat steps 3–5 two more times (for a total of three measurements).
Step 3: Measurement	Keep the step 1–5 in Step 1.
Step 4: Regular recalibration	Recalibrate regularly to keep the BP value correctly. If the wearer of the smartwatch is changed or the wearing position is changed to the other arm, the watch must be recalibrated.

In addition, the accuracy may be further reduced in patients with several medical conditions: aortic valve insufficiency with wide pulse pressure, atrial fibrillation with considerable beat-to-beat variability, peripheral vascular disease with weak perfusion, diabetes, cardiomyopathy, ESRD, neurological disorders such as hand tremor, blood clotting disorder, or taking antiplatelet agents / anticoagulants. It is also not recommended for pregnant women as the vascular characteristics differ from those of the general population due to the large hormonal changes (sudden and dramatic increases in estrogen & progesterone) during pregnancy [18].

In summary, the adequate training of BP self-measurement by standard method summarized in Table 2 is essential part for the accuracy. BP measurement using a smartwatch device is not recommended in patients with several medical conditions, high SBP ≥ 160 mmHg, or low BP ≤ 60 mmHg.

## Part 3. Gaps in the evidence and need for further studies

Currently, the purpose of BP measurement using a smartwatch device is likely to increase awareness of BP and possibly to detect HTN early in the general population, rather than to monitor treatment response in hypertensive patients. Because the current standard of AAMI validated device includes a calibration process, for the time being, accessibility to those devices seems to be low in the older generations. In contrast, considering that low awareness rate of HTN in those in the 30s to 40s age groups who may be more accustomed to those devices, smartwatch-based BP measurement can offer an opportunity to make young adults pay attention to high BP and start HTN treatment early.

However, it is well expected that hypertensive patients will use smartwatch devices for BP monitoring either in resting conditions or stress conditions. There is still scanty evidence in the use of a smartwatch device for hypertensive patients. Indeed, recent smartwatch devices fulfilled the requirement for a medical device in ISO 81060-2:2018 Non-invasive sphygmomanometers, so-called AAMI standard [11]. Although the clinical study enrolled the subjects whose gender, age, and ethnicity distribution were well satisfied with ISO criteria, only 30% of the subjects were hypertensive, and the measurement error increase according to BP increase. Therefore, the feasibility of the smartwatch device use in HTN management should be tested in many steps of clinical studies.

In terms of “usual BP” controversy, it could be reconsidered if a new standard for the validation of the smartwatch device to measure BP in an ambulatory or daily living setting is a need. In other words, when a device calibrated at resting condition, which is supposed to be used in an ambulatory setting, the accuracy of BP measured in an ambulatory setting may not be extrapolated from the calibration data. Because the reference value for the validation of a BP measuring device is only for the resting status regardless of invasive or non-invasive methods, how ambulation or non-resting condition would have impacts on the calibration parameters for smartwatch needs to be incorporated to the resting calibration data if it is possible to quantify the degree of ambulation or non-resting states. Alternatively, we might use the reference value established during the ambulatory setting, even though it does not seem feasible. In that case, it could be applied to the validation of a smartwatch to measure BP during daily living directly with the big data-based calibration to overcome the increased number of calibration parameters in various ambulatory situations. Therefore, the consensus on the need for new validation reference for smartwatch devices and its development will have a big impact on BP measurement and its related research fields.

Likely, the use of self-monitoring for the routine evaluation of hypertensive patients will be accelerated using the smartwatch devices. This trend may be recommended as part of a general movement in which patients play an increasingly important role in the management of their health. Poor adherence to therapy has been recognized as one of the most critical factors contributing to uncontrolled HTN. Knowing patients’ BP for themselves will be the most important way of improving patient’s adherence to lifestyle changes and/or medical treatment. In contrast, if patients titrated their medication based on the inadequate BP measurement, there might be harms such as doubling or skipping a dose. Importantly, self-monitoring of BPs were not evenly distributed in 24 h or 7 days, or even seasons [33]. Especially there are concerns that high BP values measured by inaccurate methods might result in unnecessary psychological stress, false-labeling of HTN, and further overspending on medical expenses [34]. The cost-effectiveness analysis of BP measurement using a smartwatch is necessary at some point.

However, BP measurements using a smartwatch might open a new field of dynamic evaluation of BP. Although absolute BP values appear to be the most important factors determining prognosis, BP variability (BPV) has also been proven in many studies to be an independent and robust indicator of a future cardiovascular events [35]. However, conventional BP measurement, even including ambulatory blood pressure measurement, mainly focused on the resting state’s static BP measurement. However, the smartphone / smartwatch-based BP measurement will naturally report BP change in daily living and physical/emotional stress, thus opening a new chapter of BP research regarding dynamic BP change. BPV is higher during weekdays and winter season, supporting that environmental factors such as job stress and the outside environment influence BPV [33]. With a more comprehensive application of smartphone / smartwatch-based cuffless BP measurement, the research about BPV in dynamic conditions will be brisk.

In summary, current status of BP measurement using a smartwatch device is for increasing awareness of BP and possibly detecting HTN early in the general population. And it is still not recommended to use BP measurement using a smartwatch device for monitoring treatment response in hypertensive patients.

## Conclusion

Smartphone technology has spread rapidly around the globe. Moreover, BP measurement using a photoplethysmography-based smartphone algorithm paired with a smartwatch will continue to grow in the foreseeable future. No one can be irresistible against this flow of the times. Recently, BP measurement using a photoplethysmography-based smartphone algorithm paired with a smartwatch is approved as a medical device, fulfilled ISO standards. The current results showed acceptable accuracy in the study population. However, there is still a considerable discrepancy in high or low BP ranges than the conventional BP measurement. The use of smartwatch-based BP measurement can improve HTN awareness, and especially in the younger population. However, there is still scanty evidence in the use of smartwatch devices for hypertensive patients. The convenience and easy-to-use is the motto of the smartphone / smartwatch device; however, it might be a ‘double-edged sword’ if the measurement were not correctly performed. Therefore, adequate education of the BP measurement method is essential to maximize benefit. Future efforts (and collaborations) should also be made by both researchers and companies to evaluate the effectiveness and usability of medical devices for the hypertensive population.

## Data Availability

Published medical data mostly of last 5 years.
